# Knowledge and practice towards care and maintenance of peripheral intravenous cannula among nurses in Chitwan Medical College Teaching Hospital, Nepal

**DOI:** 10.1002/nop2.288

**Published:** 2019-04-15

**Authors:** Chadani Osti, Menuka Khadka, Deepa Wosti, Ganga Gurung, Qinghua Zhao

**Affiliations:** ^1^ Department of Nursing Chongqing Medical University First Affiliated Hospital Chongqing China; ^2^ Department of Nursing Kathmandu Valley Hospital Kathmandu Nepal; ^3^ College of Nursing Chitwan Medical Collage Bharatpur Nepal

**Keywords:** knowledge, nurses, peripheral intravenous cannula, practice

## Abstract

**Aim:**

The study is mainly concerned about the care and maintenance of peripheral intravenous cannulation: to determine the knowledge and practice of nurses towards care and maintenance of IV cannula and to find out the obstacles encountered in caring and maintaining IV cannula. Intravenous cannulation is a common procedure performed by nurses in every hospital and closely associated with the risk of nosocomial infections if standard care is not provided.

**Design:**

A descriptive cross‐sectional study design was carried out.

**Methods:**

Nurses' knowledge and practice towards care and maintenance of peripheral intravenous cannula were assessed using a validated semi‐structured self‐administered questionnaire through the census method. Data were analysed through SPSS program. The comparison was done between knowledge and practice.

**Results:**

The findings revealed that 84.72% respondents were doing correct practices despite the fact that only 82.47% respondents had proper knowledge. Most nurses have good knowledge of caring and maintaining peripheral intravenous cannulation but there were some without proper knowledge and practice. This could be a potential risk factor for patient safety.

## 1 INTRODUCTION

### 1.1 Background

The peripheral venous catheter is the common and essential intravenous (IV) device, frequently used in medical practices (Bijayalaxmi, Urmila, & Prasad, 2010; Webster, Osborne, Rickard, & New, 2015). Peripheral intravenous cannulation (PIC) is an invasive procedure performed in hospitalized patients (Goudra, Galvin, Singh, & Lions, 2014; Urbanetto Jde, Peixoto, & May, 2016; Webster et al., 2008), where the patient's skin is punctured with a needle to allow insertion of a temporary plastic tube into a vein. It is an integral part of professional nursing practice in all the healthcare institutions (Arbaee, 2016), which is done for different purposes like IV infusion and medications (Ray‐Barruel, Polit, Murfield, & Rickard, 2014) and is kept for the different duration of time depending on patient's condition with a potential risk of microbial growth (Urbanetto Jde et al., 2016). Such infections are also the part of nosocomial infections and relatedly associated with an increase in days of hospital stay, morbidity, mortality and hospital costs (Bijayalaxmi et al., 2010; Lavery, 2011; Miller & O'Grady, 2012; Osti, Wosti, Pandey, & Zhao, 2017).

Approximately 60% of hospital inpatients annually undergo PIC to receive therapeutic IV medication. This may result in hospital‐acquired bacteraemias as 6.2% of such incidence is directly attributed to the PIC. PIC is more commonly associated with localized than systemic infection. Thrombophlebitis and infection are common complications of PIC (Arbaee, 2016). Between 2.3%–67% of patients develop thrombophlebitis (Webster et al., 2008). Between 1.5%–60% of phlebitis is associated with PIC (Webster et al., 2015). In the United States, 80,000 catheter‐related bloodstream infection (CRBSIs) and 250,000 cases of CRBSIs occur in intensive care units annually (Miller & O'Grady, 2012).

Nurses play a vital role in the prevention of such infections (Osti et al., 2017). Most of the interventions and prevention strategies such as insertion, monitoring and assessing peripheral venous catheter (PVC) site are part of routine nursing care (Arbaee, 2016). The nurse should have accurate knowledge of the preparation and administration of the IV Infusion and IV device. In addition, they should also know about the prevention, treatment and management of local and systematic complications supported by dynamic evidence‐based practice guidelines. One of the major risks for phlebitis incidence is due to the placement and maintenance of PVC by insufficiently trained staff.

### 1.2 Aim

Reflecting on the facts mentioned above, the current study is carried out to determine the knowledge and practice of nurses towards the care and maintenance of IV cannula and obstacles encountered during the procedure. Intravenous cannulation is common procedures performed by nurses in every hospital and closely associated with the risk of nosocomial infections if standard care is not provided. The results from the study can help in formulating a better programme, which reduces the incidence of PIC‐related infections and uplifts the standard of care. Following the discussion above, the questions of interest include the following:

- What are the contributing factors leading to the complication of peripheral intravenous cannulation?
- What are the implementations needed for correct nursing practices to care and maintain peripheral intravenous cannulation?

Therefore, the significance of the study will be to analyse the nurse's knowledge towards the care and maintenance of peripheral intravenous cannulation at Chitwan Medical College Teaching Hospital (CMCTH).

## 2 STUDY

### 2.1 Design

A descriptive cross‐sectional study with a quantitative approach design was used in the research. The research design incorporated both quantitative and qualitative methodologies to find out in‐depth descriptive information about care and maintenance of peripheral intravenous cannulation. Nurses' knowledge and practice towards care and maintenance of peripheral intravenous cannula and its association with experience, education, position and the department were assessed using a validated semi‐structured self‐administered questionnaire.

### 2.2 Sample and setting

Census method of sampling technique was used among the nurses of CMCTH. Only the inpatient department nurses, both the junior and the senior who were interested to participate in the study, were included, whereas those on long leave, uninterested or from outpatient department were excluded. Ultimately, two hundred nurses participated in the study from different inpatient wards.

### 2.3 Data collection

The valid and reliable standard structured tool was used for data collection, developed by Author Ahmad Nizal Mohd Ghazali and colleagues (Arbaee, 2016). The adapted validated semi‐structured self‐administered questionnaire was distributed to the selected nurses and collected after they finished on the same day.

Each questionnaire comprised four sections. The first section was about respondents' demographic characteristics, years of work experiences, education level, position and working department. Followed by the second section about knowledge on care and maintenance of peripheral intravenous catheter, which was measured through 19 premises with a normal scale (“yes,” “no” and “I don't know”). Similarly, the third section was about nurses' practice towards care and maintenance of peripheral intravenous cannulation and was measured through total 17 premises, where 16 premises with a normal scale (yes, no and I don't know) and remaining 1 with three different options. And finally, the fourth section was barriers encountered for caring and maintaining peripheral intravenous cannulation, which was analysed following the similarity of the answers.

### 2.4 Data analysis

The collected data were checked, reviewed and organized daily for its completeness and consistency. The data were entered into the statistical package for social science (SPSS) version 20 and then analysed and interpreted in terms of descriptive statistics like frequency and percentage.

### 2.5 Ethics

For ethical consideration, official permission letter was taken from CMCTH. Informed consent was taken from respondents after clarification of the objectives of the study. Respondents were assured that the information they provide would be confidential and were allowed to participate in a free and unbiased environment.

## 3 RESULTS

Demographic data like work experience, educational level, position and the working department would influence their understanding and knowledge towards care and maintenance of PIC. Findings of demographic data revealed that among 200 nurses, most respondents (57%) had work experiences below 1 year; they were still new without enough experiences. Most (76%) of their educational levels were PCL Nursing. Regarding their position, 86% were staff nurse and only 14% was a senior staff nurse. More than half of the respondents (53.5%) were in the critical unit (Table 1).

**Table 1:** Demographic characteristics of the respondents (*N* = 200)

S.NO	Variables	Categories	*N* (%)
1.	Year of work experience	Up to 1 year	114 (57.0)
		1–2 Years	56 (28.0)
		2–3 Years	12 (6.0)
		More than 3 years	18 (9.0)
2.	Educational level	PCL Nursing	152 (76.0)
		Bachelor in Nursing	48 (24.0)
3.	Position	Staff Nurse	172 (86.0)
		Senior Staff Nurse	28 (14.0)
4.	Working Department	Critical Care Unit	107 (53.5)
		General Adult Wards	59 (29.5)
		General Pediatric Ward	13 (6.5)
		Maternity Ward	10 (5.0)
		Dialysis	11 (5.5)

Note

*N* = total number of respondents.

Nurses' knowledge towards the care and maintenance of peripheral IV cannula was assessed through nineteen questions as shown in Table 2. Each question had options for selecting right, wrong or having no idea. Most (82.47%) of the respondents have proper knowledge, 13.21% of the respondents have the wrong knowledge, and 4.32% of respondents have no idea about proper care.

**Table 2:** Knowledge towards the care and maintenance of the peripheral IV cannula (*N* = 200)

S.N	Variables	Yes *N* (%)	No *N* (%)	I don't know *N* (%)
1	The cannula gauge 14−20 G is suitable in adult patient and 22−24 G in paediatric patient	200 (100.0)	—	—
2	Veins at dorsal and ventral surface of the upper extremities are used for IV cannulation	165 (82.5)	18 (9.0)	17 (8.5)
3	Peripheral IV cannula must be removed every 12–72 hr from insertion time	174 (87.0)	25 (12.5)	1 (0.5)
4	IV cannula can be used 48–72 hr if no signs and symptoms of complication	171 (85.5)	17 (8.5)	12 (6.0)
5	Phlebitis is the most identifiable infection	194 (97.0)	5 (2.5)	1 (0.5)
6	The environment sanitation influent the risk of IV infection	151 (75.5)	42 (21.0)	7 (3.5)
7	Hand hygiene before IV cannula insertion prevents infection	197 (98.5)	3 (1.5)	—
8	Maintaining aseptic technique only during IV insertion helps to prevent infection	73 (36.5)	123 (61.5)	4 (2.0)
9	Wearing non‐sterile gloves during IV cannula insertion is advisable	159 (79.5)	36 (18.0)	5 (2.5)
10	Skin preparation at insertion site is essential	186 (93.0)	13 (6.5)	1 (0.5)
11	Increase attempts for cannulation will increase the risk of infection	166 (83.0)	21 (10.5)	13 (6.5)
12	Transparent dressing will help to recognize early signs and symptoms of infection	134 (67.0)	39 (19.5)	27 (13.5)
13	Removing extra IV cannula will help to reduce risk of infection occur	170 (85.0)	29 (14.5)	1 (0.5)
14	*Staphylococcus aureus* is the most associated with cannula tips	128 (64.0)	32 (16.0)	40 (20.0)
15	Catheter material, size, duration, experience of the staff etc. influences on risk of infection	181 (90.5)	14 (7.0)	5 (2.5)
16	IV therapy increases risk of IV infection	136 (68.0)	48 (24.0)	16 (8.0)
17	Patient with PIC is on risk of nosocomial infection	139 (69.5)	49 (24.5)	12 (6.0)
18	Patient education on care of IV cannula is important to reduce risk of infection	175 (87.5)	23 (11.5)	2 (1.0)
19	I/V cannula should be flushed by inj NS after any IV Medication	185 (92.5)	15 (7.5)	—

Note

*N* = total number of respondents.

Table 3 shows the respondent's practice on care and maintenance of IV cannula. Here, sixteen statements were given to assess their practice. Most (84.72%) of the respondents followed the proper practice, 14.22% respondent did not follow the proper practices, and minority (1.06%) were not confident on their practice whether they were doing correct or incorrect.

**Table 3:** Nursing practice on the care and maintenance of the peripheral IV cannula (*N* = 200)

S.N	Variables	Yes *N* (%)	No *N* (%)	I don't know *N* (%)
1	I always change IV cannula after 72 hr	174 (87.0)	24 (12.0)	1 (0.5)
2	I immediately change site if sign of phlebitis	196 (98.0)	2 (1.0)	2 (1.0)
3	I always use transparent IV dressing	—	200 (100.0)	—
4	I always proper documentation	167 (83.5)	32 (16.0)	1 (0.5)
5	I use administration set for 72 hr only	167 (83.5)	22 (11.0)	11 (5.5)
6	I aware of complications of IV cannulation	197 (98.5)	2 (1.0)	1 (0.5)
7	I always maintain aseptic technique	199 (99.5)	1 (0.5)	—
8	I always change the dressing when it wet	190 (95.0)	10 (5.0)	—
9	I always educate my patient about IV care	166 (83.0)	34 (17.0)	—
10	I always teach patient about IV infection	177 (88.5)	23 (11.5)	—
11	I aware of hand hygiene before insertion	197 (98.5)	3 (1.5)	—
12	I aware of skin preparation before insertion	189 (94.5)	7 (3.5)	4 (2.0)
13	I aware the risk factors of infection	191 (95.5)	6 (3.0)	3 (1.5)
14	I always follow guidelines for IV cannulation	185 (92.5)	7 (7.0)	1 (0.5)
15	I am confident enough to carried out IV cannulation procedure	155 (77.5)	36 (18.0)	9 (4.5)
16	I always do IV flush by Inj NS after IV medication through cannula	160 (80.0)	39 (19.5)	1 (0.5)

Note

*N* = total number of respondents.

In Table 4, nurses' intervention if no signs and symptoms of complication or infection after 72 hr of IV cannula insertion was assessed, where most respondents changed the new cannula.

**Table 4:** Nursing interventions if there is no signs and symptoms of the complication or infection even after 72 hr of the IV cannula insertion (*N* = 200)

S.N	Interventions	*N* (%)
1	Leave the cannula in situ	26 (13.0)
2	Reset the new IV cannula	136 (68.0)
3	Record and hand over to continue assessment	38 (19.0)

Note

*N* = total number of respondents.

In Table 5, barrier encountered in caring and maintaining of peripheral IV cannulation was asked with respondents. Most respondents agreed on the incooperated patient and small vein prone to blockage and damage. The minorities agreed on giving too strong medication make the vein easily block.

**Table 5:** Barrier encountered in caring and maintaining of the peripheral IV cannula (*N* = 200)

S.N	Encountered barrier	Yes *N* (%)	NO *N* (%)
1	Incooperated patient	61 (30.5)	139 (69.5)
2	Give too strong medication make the vein easy block	40 (20.0)	160 (80.0)
3	Small vein prone to blockage and damage	116 (58.0)	84 (42.0)

Note

*N* = total number of respondents.

## 4 DISCUSSION

The current study assesses the knowledge and practices regarding caring and maintaining PIC among the nurses of CMCTH, Bharatpur, Nepal. This study found that most nurses have good knowledge of IV cannula protocols and are also doing proper practice. Lack of knowledge of infection prevention and proper nursing care among nurses may become a barrier in adhering to evidence‐based guidelines for preventing IV catheter‐related infections. Despite the known complications of PICs, there are still nurses who are not practicing the standard protocols and are undertaking an incorrect way of insertion, removal as well as maintenance of IV cannula.

The Centers for Disease Control (CDC) also published the second edition of Guidelines for the Prevention of Intravascular Catheter‐Related Infections on 9 August 2002, replacing its original guideline published in 1996. The goal was to provide evidence‐based recommendations for preventing catheter‐related infections (O'Grady et al., 2011). Major areas of emphasis in the 2002 CDC Guidelines included educating and training healthcare providers who insert and maintain catheters; using maximal sterile barrier precautions; using a 2% chlorhexidine preparation for skin antisepsis; avoiding routine replacement of central venous catheters as a strategy of infection prevention; and using antiseptic or antibiotic impregnated short‐term central venous catheters and chlorhexidine‐impregnated sponge dressings if the rate of infection is high despite adherence to other strategies (i.e., education and training, maximal sterile barrier precautions and 2% chlorhexidine for skin antisepsis) (Miller & O'Grady, 2012; O'Grady et al., 2011). The 2002 CDC guideline now has been revised and updated again and published in 2011 (O'Grady et al., 2011), and it was prepared by a working group comprising members from professional organizations representing the disciplines of critical care medicine, infectious diseases, healthcare infection control, surgery, anaesthesiology, interventional radiology, pulmonary medicine, paediatric medicine and nursing (Miller & O'Grady, 2012).

Choosing the IV route and selecting an appropriate size of vascular access device are the most important factors in preventing IV site infection (Scales, 2008). In this study, 100% of respondents have knowledge of the appropriate size of the cannula. For site selection, the PIC is usually inserted into a metacarpal vein on the back of the hand or a vein in the lower arm, either the cephalic or basilic vein. However, femoral veins should be avoided because of the higher density of skin flora in this area, which would put the patient at increased risk of infection (Arbaee, 2016; Scales, 2008). In this study, most respondent (82.5%) knew about the site selection for IV cannulation, whereas remaining 17.5% respondent did not know. Scheduled replacement of catheters is proposed as a means to prevent phlebitis and catheter‐related infections. And when the catheter site dressing becomes loose, wet or soiled, an aseptic non‐touch technique dressing should be performed (O'Grady et al., 2011).

Many interventions have been developed to reduce the incidence of phlebitis such as including new catheter materials and innovative methods for securing the catheter but the most widely practiced intervention is the routine replacement of the catheter (Li, Liu, & Qin, 2016; Webster et al., 2008, 2015; Dougherty, 2000). The CDC guidelines also recommend that peripheral intravenous catheter should be removed or replaced every 12–72 hr to avoid complication such as thrombophlebitis (Arbaee, 2016; Miller & O'Grady, 2012; O'Grady et al., 2011). Most hospitals in China also follow this CDC recommendation (Li, Liu, & Qin, 2016; Dougherty, 2000). The two major sources of bloodstream infection associated with IV device are colonization of the device itself and contamination of the fluid administered through the device (Bijayalaxmi et al., 2010). About 87% of the respondents in this study also knew as well as followed the practice of removing the IV cannula in every 12–72 hr from insertion. Also, most respondents (98%) immediately changed the IV cannula to the non‐infected part when they saw the sign of phlebitis.

Nurses' knowledge and early recognition of risk factors for the development of phlebitis can reduce complications, which improves the quality of care, patient safety, patient satisfaction ratings and at the same time reduces the length of hospital stay and the overall cost of health care (Milutinovic, Simin, & Zec, 2015). In this study, 97% of respondents have knowledge that thrombophlebitis and infection are the common complications of IV cannulation. Similarly, 75.5% of respondents were aware of the influences of environmental cleanliness on IV site infection.

The CDC provides guidelines for protection against infection of the peripheral catheters which includes good hand hygiene before catheter insertion or maintenance either through the use of waterless, alcohol‐based product or an antibacterial soap and water with adequate rinsing, along with proper aseptic technique during catheter manipulation (Miller & O'Grady, 2012; O'Grady et al., 2011; World Health Organization, 2009). Hand washing is the cost‐effective measures to minimize nosocomial infection (Osti et al., 2017; World Health Organization, 2009). In this study also, almost the entire respondent (98.5%) knew the importance of hand hygiene before IV insertion. But only 61.5% of the respondent believed maintaining aseptic technique only during insertion of IV cannula would not prevent infection. However, 79.5% of the respondents agreed on wearing non‐sterile gloves during insertion of IV cannula. And, 93% of the respondents knew skin preparation at the insertion site is required before IV cannula insertion. Almost all respondents (99.5%) always maintained aseptic technique during preparing, inserting and removing of IV cannula and were aware of the factors that influence the risk of infection. About 94.5% of respondents were aware of the importance of skin preparation before the procedure. Only 83% of respondents knew increased attempts for cannulation would increase the risk of infection.

Transparent dressings consistently secure the device, permit continuous visual inspection of the catheter site, permit patients to bathe and shower without saturating the dressing and require less frequent changes than do the tape dressings. It can be safely left on PIC for the duration of catheter insertion without increasing the risk for thrombophlebitis (O'Grady et al., 2011). An inadequately secured PIC also increases the risk of CRBSIs, as the pistoning action (moving back and forth in the vein) of the catheter can allow migration of organisms along the catheter and into the bloodstream (Marsh, Webster, Mihala, & Rickard, 2015). This information was known to only 67% who also failed to use transparent dressing in practice. Additionally, 95% of respondents changed the dressing when it was wet or dislodged. Removing IV cannula immediately when not in use helps in reducing the risk of infection occurrence, which was agreed by 85% of the respondents. Accurate documentation like the date and time of cannula insertion, labelling IV equipment and fluid containers with date and time they are opened to ensure they have changed appropriately demonstrate better cannula care, encourage research‐based standardized practice and provide guidance as well as evidence of competence (Scales, 2008; Trim, 2005). 83.5% of respondents were found doing proper documentation of the IV insertion. Similarly, 83.5% of respondents used administration set for IV cannula within 72 hr and 98.5% respondents were aware of complications of IV cannulation for instance infiltration, phlebitis and extravasation. Meanwhile, 68% of respondents were aware that giving intravenous therapy would increase the risk of infection through a peripheral IV catheter. In this study, 69.9% of the respondents knew patients were on high risk to get a nosocomial infection when receiving IV therapy. But only 64% of respondents had knowledge that *Staphylococcus aureus* is the most associated with cannula tips. Nevertheless, 90.5% respondents had knowledge that factors like catheter material, catheter size, catheter movement, an experience of the staff, duration of catheterization, composition of infusate and frequency of dressing change would influence the risk of infection occurrence. Educating patients on how to care IV cannula also helps to reduce the risk of infection, which was known to 87.5% of respondents but 88.5% of respondents educated their patient. 92.5% of respondents know IV cannula should be flushed by inj NS after any IV Medication but only 80% follow this. Among 200 respondents, 92.5% always follow guidelines of IV cannulation given by the hospital management. And, only 77.5% of respondents were confident enough to carry out the IV cannulation procedure.

Nurses were also asked what intervention they do if no signs and symptoms of complication or infection occurred even after 72 hr of IV cannulation. Among them, most respondents (68%) reset the new IV cannula, 13% of respondents left the cannula in situ, and 19% of respondents record and hand over to continue assessment. Regarding barrier encountered in caring and maintaining of peripheral IV cannulation, 30.5% of respondents stated it was due to the incooperated patient, 20% of respondents told it was due to strong medication blocking the vein, and 58% of respondents stated that small veins were prone to blockage and damage.

### 4.1 Limitation and recommendation

This study is undertaken in a single hospital and is only analysed quantitatively. The scope of the study can be increased to several hospitals without limiting only to quantitative design but also including qualitative study design for assessing the factors affecting the nurses' knowledge and practices. This may help in better generalizations of the findings, which can be used to reduce hospital‐acquired infections related to the PIC and to enhance the quality of care in the hospital.

## 5 CONCLUSION

The risk and complications of PIC could endanger the patient's life. So, in the clinical area, nurses must be knowledgeable and competent in every aspect of IV cannulization. In this study, most nurses were having a good knowledge of caring and maintaining of peripheral IV cannulation but there were still some nurses who did not have proper knowledge and experience for using IV cannulation which could be a potential risk factor for patient safety. This may be attributed to the fact that most respondents were junior nurses with <1 year experience in the clinical area. Their knowledge towards care and maintenance of IV cannula was very limited which might result in practicing incorrect method. The results should sensitize healthcare managers to improve nursing training and education, according to clinical risk management perspectives.

## CONFLICT OF INTEREST

None.

## AUTHOR CONTRIBUTIONS

CO: study conception and design, data collection, literature reviews, analysis and drafting of the manuscript. GG: data collection. MK, DW and QZ: data analysis, literature review and drafting of the manuscript.
